# Positive Behaviors and Strengths of People With Dementia

**DOI:** 10.1093/geroni/igaa057.883

**Published:** 2020-12-16

**Authors:** Diana DiGasbarro, Courtney Whitaker, Benjamin Mast

**Affiliations:** 1 University of Louisville, Louisville, Kentucky, United States; 2 Masonic Homes Kentucky, Louisville, Kentucky, United States

## Abstract

Assessment of and care for people with dementia is shifting away from a purely medical disease model toward a more holistic, biopsychosocial approach. Within this movement is growing interest in balancing the negative, symptom-based view of people with dementia with better understanding of strengths and positive behaviors exhibited by people with dementia. The aim of this qualitative study was to gather perspectives of professional caregivers regarding positive behaviors and strengths observed in people with dementia. Data were obtained from three focus groups conducted at a memory care unit within a large long-term care facility. Focus group participants (N=14) worked in nursing and the activities department. Inductive methods were used to code and analyze focus group transcripts and recordings. Five major themes were identified: enduring abilities and values, enduring traits and strengths, sense of purpose and meaning, desire to be helpful, and prosocial behavior. These themes illustrate many domains in which professional caregivers have observed positive behaviors and strengths in people with dementia living in a long-term care facility. The results of this study contribute to the growing literature pertaining to the intersection of positive psychology and dementia research and practice. Future directions include development of an assessment tool to measure positive behavior and strengths in people living with dementia.

